# Red Blood Cell Deformability Is Expressed by a Set of Interrelated Membrane Proteins

**DOI:** 10.3390/ijms241612755

**Published:** 2023-08-13

**Authors:** Gregory Barshtein, Alexander Gural, Dan Arbell, Refael Barkan, Leonid Livshits, Ivana Pajic-Lijakovic, Saul Yedgar

**Affiliations:** 1 Department of Biochemistry, The Faculty of Medicine, The Hebrew University of Jerusalem, Jerusalem 9112001, Israel; gregoryba@ekmd.huji.ac.il; 2 Blood Bank, Hadassah University Hospital, Jerusalem 9112001, Israel; gural@hadassah.org.il; 3 Pediatric Surgery, Hadassah University Hospital, Jerusalem 9112001, Israel; arbell@hadassah.org.il; 4 Department of Digital Medical Technologies, Holon Institute of Technology, Holon 5810201, Israel; refaelb@hit.ac.il; 5 Red Blood Cell Research Group, Vetsuisse Faculty, Institute of Veterinary Physiology, University of Zurich, 8057 Zürich, Switzerland; leonidlivshts@gmail.com; 6 Department of Chemical Engineering, University of Belgrade, 11120 Belgrade, Serbia; iva@tmf.bg.ac.rs

**Keywords:** red blood cells, RBC deformability, membrane proteins, lipid rafts, membrane vesicles, membrane remodeling

## Abstract

Red blood cell (RBC) deformability, expressing their ability to change their shape, allows them to minimize their resistance to flow and optimize oxygen delivery to the tissues. RBC with reduced deformability may lead to increased vascular resistance, capillary occlusion, and impaired perfusion and oxygen delivery. A reduction in deformability, as occurs during RBC physiological aging and under blood storage, is implicated in the pathophysiology of diverse conditions with circulatory disorders and anemias. The change in RBC deformability is associated with metabolic and structural alterations, mostly uncharacterized. To bridge this gap, we analyzed the membrane protein levels, using mass spectroscopy, of RBC with varying deformability determined by image analysis. In total, 752 membrane proteins were identified. However, deformability was positively correlated with the level of only fourteen proteins, with a highly significant inter-correlation between them. These proteins are involved in membrane rafting and/or the membrane–cytoskeleton linkage. These findings suggest that the reduction of deformability is a programmed (not arbitrary) process of remodeling and shedding of membrane fragments, possibly mirroring the formation of extracellular vesicles. The highly significant inter-correlation between the deformability-expressing proteins infers that the cell deformability can be assessed by determining the level of a few, possibly one, of them.

## 1. Introduction

The primary role of red blood cells (RBC) is to supply oxygen to tissues. To accomplish this, RBCs have unique flow-affecting properties [1,2], which define hemodynamic functionality, namely their capacity to affect blood circulation [3]. A major effector of the RBC hemodynamic functionality is the cell’s deformability, expressing the cells’ ability to adapt their shape to the dynamically changing flow conditions to minimize their resistance to flow. This is particularly important for their passage through the capillaries, which are narrower than the RBC. Reduced deformability (increased rigidity) results in impaired perfusion and oxygen delivery to peripheral tissues [4,5,6], and rigid RBCs can directly block capillaries [7]. RBC deformability is also a significant determinant of their ability to pass through the splenic vasculature; reduced deformability hinders their transit and increases splenic RBC sequestration and destruction [8,9,10,11]. Accordingly, reduced RBC deformability (increased rigidity) has been implicated in circulatory disorders and anemias observed in diverse pathologies, e.g., thalassemia, sickle anemia, cerebral malaria, sepsis, diabetes [1,12,13,14,15], cardiovascular conditions, and stroke [4,16]. A reduction in deformability is characteristic of RBC physiological aging, and normally the deformability distribution spans from low-deformable, even undeformable cells, to highly deformable cells [2,17,18].

The aging of RBCs that occurs during their circulation in the vascular system is associated with metabolic and structural changes [19] that lead to remodeling of the cell membrane and alteration of cytoplasm composition [20,21]. At the cellular level, aging is characterized by decreased cell volume (MCV) due to the shedding of cell membrane fragments [22,23], leading to the elevation of the intracellular hemoglobin (Hb) concentration (MCHC) and cell density [24].

Shedding membrane fragments is associated with reorganizing the RBC cytoskeletal and losing membrane lipids and proteins [25,26], resulting in RBCs with a reduced area-to-volume ratio and decreased cell deformability.

Currently, RBC deformability is determined exclusively by physical methods, using various techniques, such as micropipette aspiration [27], atomic force microscopy [28], optical tweezers [29,30], filtration [31], microfluidic filtration [32], laser diffractometry [33], erythrocyte shape recovery [34], and direct visualization of the cell shape change under flow in the flow chamber [2,17,35,36]. However, the cell’s physical properties are determined by its structure and composition, and their alteration should correlate with changes in biochemical measures.

The present study was undertaken to explore the relation of RBC membrane protein composition to cell deformability. To this end, we comprehensively analyzed the correlation of the RBC membrane proteins, determined by mass spectroscopy (MS), with the cell deformability, as determined by image analysis. It was found that the change in the RBC deformability is associated with changes in the level of a set of interrelated membrane proteins.

## 2. Results

Correlation between RBC deformability and the level of membrane proteins.

The MS analysis identified 752 membrane proteins, and all were examined for correlation with the RBC deformability expressed by the cell elongation ratio (ER). 

As shown in Table 1, fourteen membrane proteins exhibited a clear positive correlation with cell deformability; the higher the protein level, the higher the cell deformability.

**Figure 1:**
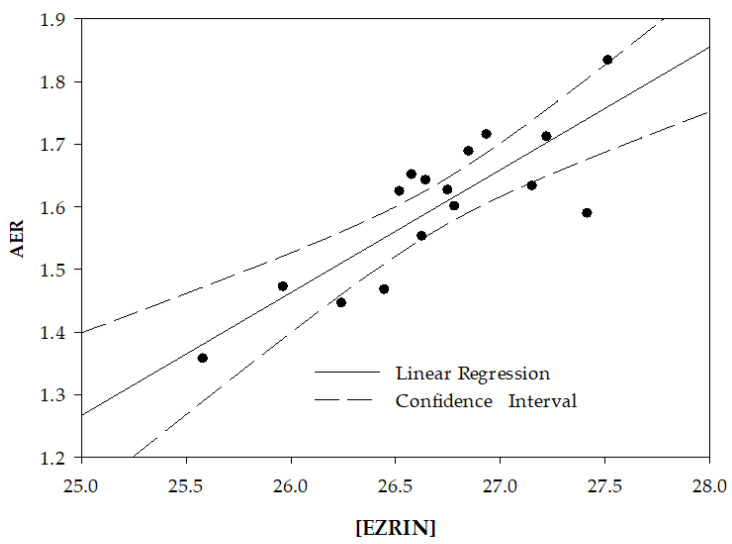
Correlation between the level of ezrin (expressed by the value of Ln (LFQ)) in RBC membrane and cell deformability, expressed by the average elongation ratio (AER). r = 0.83; *p* = 0.00006.

The relationship between the AER value and the content of cell membrane proteins is further illustrated in Figure 1, depicting the linear regression between AER and the level of ezrin in the membrane, which exhibits the highest correlation significance (Table 1).

### 2.1. Variability in RBC Deformability and Respective Level of Membrane Proteins

**Figure 2:**
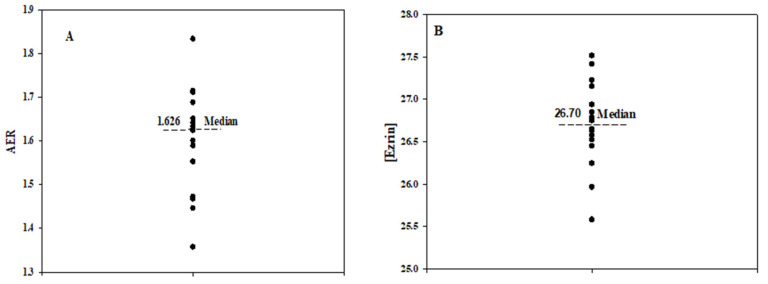
Distribution of the deformability (AER, (**A**)) and the membrane ezrin level (**B**) in the RBC samples.

Following our previous study [17,18], the RBC deformability exhibited large variability between the samples regardless of their source, where the average elongation ratio (AER) ranged from 1.36 to 1.83, as shown in Figure 2A.

Similarly, considerable variability was found in the level of the deformability-expressing membrane proteins listed in Table 1. This is illustrated in Figure 2B, which presents the distribution of membrane ezrin content (Ln (LFQ)) in the tested RBC samples, ranging from 25.6 to 27.5.

### 2.2. Interrelation between the Deformability-Expressing Membrane Proteins

The changes in the deformability-expressing membrane proteins can occur independently for the individual proteins or inter-dependently. We analyzed the correlations between the protein levels listed in Table 1 to gain insight into this question. As shown in the matrix of Table 2, a highly significant mutual inter-correlation was found between the deformability-expressing proteins.

The clear inter-correlations between the deformability-expressing proteins suggest that, in practice, the cell deformability can be assessed by determining the level of a few, or even one of them, particularly the ones that exhibit especially strong correlation such as ezrin, band-3, flotillin-1, stomatin, flotillin-2, glycophorin C, protein 4.1, and CD44. 

**Figure 3:**
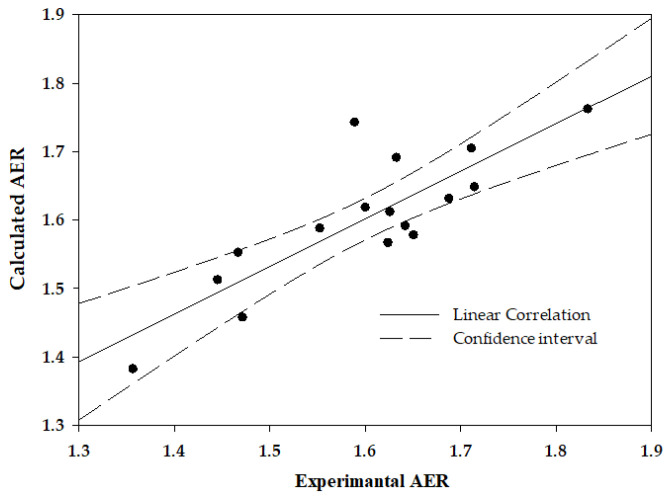
RBC deformability, expressed by AER measured directly by the image analysis (by the CFA) vs. AER calculated by the cell membrane ezrin level. r = 0.83, *p* = 0.00006.

This is illustrated in Figure 3, showing the correlation between the deformability measured directly by the image analysis (CFA) vs. AER calculated by the levels of ezrin, following one-parametric linear regression AER = 0.1964 × [EZR] − 3.642.

Table 2 shows that the fourteen proteins can be divided into two groups according to the significance of the inter-dependence between their membranal content. 1. The twelve proteins are characterized by a very high value of the Person correlation coefficient (r > 0.775) of inter-dependences. 2. The two proteins with r < 0.775 are ezrin and G-adducin. We tested the possibility of achieving a better correlation between EAR’s experimental and calculated values on these grounds. To this end, we derive a two-parametric linear regression in which we combined one protein from the first and one protein from the second group. The best correlation between the experimental and calculated AER was given from all the regressions when protein 4.1 (Group 1) and ezrin (Group 2) were used. Yielding the regression equation is AER = 0.129 × [EZR] + 0.066 × [EPB41] − 4.09, where [EZR] and [EPB41] are the membrane content of ezrin and protein 4.1R (expressed by the value of Ln (LFQ)).

**Figure 4:**
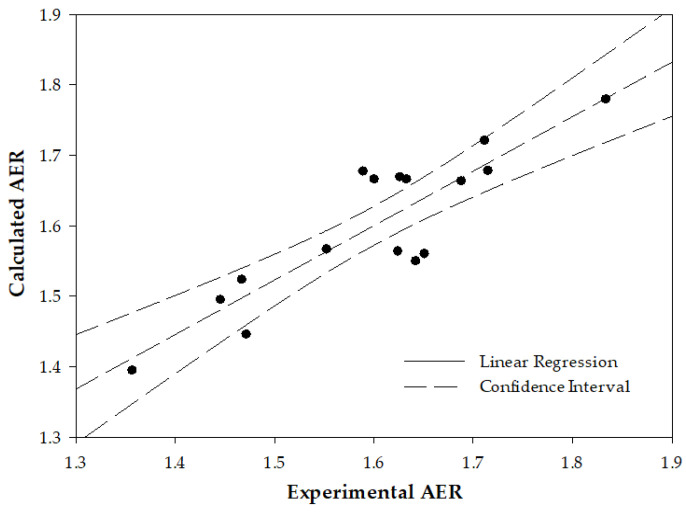
RBC deformability, expressed by AER, measured directly by image analysis (using the CFA) vs. AER calculated by the combined levels of ezrin and protein 4.1 in the cell membrane (using a two-parametric linear regression model). r = 0.877, *p* = 0.00006.

The predictive power of this model is illustrated in Figure 4, showing a better correlation between the AER derived from the combined calculation of these proteins’ levels vs. the AER measured directly by the image analysis; r = 0.877, significance, *p* = 0.00006.

It should be noted that the two-parameter model is not ideal due to the inter-correlation between the content of the two proteins. To minimize this drawback, in Figure 4, we chose the two proteins with the lowest inter-correlation (ezrin and protein 4.1).

## 3. Discussion

As a mechanical property, RBC deformability depends on the cell membrane structure and composition, and their alteration should thereby correlate with changes in biochemical measures. Previous studies, both experimental and numerical simulations, linked the cell deformability to the content of specific membrane proteins, particularly stomatin, band-3, and protein 4.1R [37,38,39,40].

The present study is the first to comprehensively analyze the changes in levels of RBC membrane proteins and their potential relation to cell deformability.

Out of the 751 RBC membrane proteins that were identified, only 14 exhibited a strong positive correlation with the cell deformability, including two groups of proteins with known functions: (1) Proteins involved in the linking of the lipid bilayer and the cytoskeleton, specifically band-3, protein 4.1, glycophorin C, an ezrin adducin [41,42], and (2) proteins involved in membrane lipid raft, specifically stomatin, flotillin-1, and flotillin-2 [26,43,44,45]. Most of these proteins are lost by the cell membrane during its physiological aging [46,47], which is also known to be accompanied by decreased deformability [48].

The main three determinants of red cell deformability are (a) the surface area-to-volume ratio of a cell [49]; (b) intracellular viscosity [50]; and (c) the membrane viscoelasticity [10]. Of these three factors, only the elasticity of the membrane is directly determined by its protein composition, which is involved in linking the membrane lipid bilayer with the cytoskeleton [51,52,53,54].

The erythrocyte membrane is a two-dimensional structure consisting of a cytoskeleton and lipid bilayer with integral membrane proteins. The RBC cytoskeleton network predominantly comprises spectrin tetramers, actin, and protein 4.1R, along with adaptor proteins, attached to the membrane by band-3 via ankyrin [55,56]. Disruption of the bilayer and skeleton contact alters the cells’ shape and mechanical properties [10,54]. RBC ghosts with an impaired membrane–cytoskeleton attachment have a significantly lower surface area and volume and, respectively, lower than normal deformability [57].

This is consistent with the reduction in the level of the deformability-expressing proteins presented in Table 1, which, as noted above, most of them are involved, to various degrees, in the link between the lipid bilayer and the cytoskeleton or are a part of the lipid rafts.

The highly significant inter-correlation between the deformability-expressing proteins, depicted in Table 2, infers that the reduction in their level is a programmed (not arbitrary) process of membrane remodeling and possibly shedding of membrane fragments. Our previous study showed that the RBC deformability is inversely proportional to the number of microvesicles in the extracellular medium [48]. It was also shown that some of the deformability-expressing proteins listed in Table 1 (stomatin, band-3, flotillin-1, and flotillin-2; ezrin, protein 4.1, and argonaute-2) are integral components of the vesicles formed by the red cell [26,58,59,60], implying that the vesiculation is accompanied with a decrease in the levels of these proteins in the cell membrane [45,56,61,62]. 

Notably, a number of the deformability-expressing proteins are included in the “stomatin complex” [63], and we have previously shown the association between the RBC deformability and the cell membrane stomatin level [25]. 

It is thus plausible to conclude that membrane remodeling, which reflects the decrease in cell deformability, involves the shedding of membrane fragments and the concomitant formation of microvesicles. 

It should be noted that the deformability-expressing proteins listed in Table 1 do not include some proteins that are known to be involved in the connection between the cytoskeleton and the lipid bilayer of the red cell, e.g., moesin, ankyrin, and cofilin [64,65]. Thus, the change in the cell deformability would also be associated with changes in these proteins’ levels. This discrepancy is yet to be explored. 

As noted in the Introduction, RBC deformability, a mechanical property of the cell, is commonly determined by physical methods. The study presents a set of membrane proteins whose determination by biochemical methods (immunochemistry, mass spectroscopy) can be used for this purpose. Moreover, this study presents a highly significant inter-correlation between the deformability-expressing proteins (Table 2), as well as an excellent correlation of ezrin vs. deformability (Figure 1), and between the calculated vs. experimentally determined deformability, using one or two proteins (Figure 3 and Figure 4, respectively). This suggests that the cell deformability can be assessed by determining a few, or possibly one, of the deformability-expressing proteins. This can be performed by chemical methods, e.g., immunochemistry, mass-spec, and similar, which might be either less efficient or more efficient than the physical methods for measuring RBC deformability, depending on the specific methods.

It should be noted that the above changes in the RBC membrane composition leading to alteration of the cell deformability can be different in pathological conditions associated with alterations in RBC composition and structure, such as hemoglobinopathies and diabetes [66,67,68], and should hopefully be the subject of further studies.

## 4. Materials and Methods

### 4.1. Materials

Phosphate-buffered saline (PBS) without calcium and magnesium (catalog 02-023-1A) was purchased from Biological Industries Ltd. (Kibbutz Beit-HaEmek, Haemek, Israel). All other chemicals were purchased from Sigma Aldrich Israel.

### 4.2. Methods

#### 4.2.1. RBC Sample Sources

Sixteen RBC samples were obtained from two sources: 1. Freshly collected blood from healthy volunteer donors (male, 18–40 years, Hb > 13 mg/dL), without known disorders, upon their consent; 2. expired packed RBC (PRBC) units (stored for 42 days). All blood samples were taken according to the guidelines and approval of the Helsinki Committee Regulations, Hadassah Hospital, Jerusalem, Israel. Permit 0819-20-HMO.

#### 4.2.2. Isolation of RBC

Healthy volunteers: Fresh blood was drawn from ten healthy donors. RBCs were isolated and washed twice in PBS by centrifugation (500× *g* for 10 min) and re-suspended in PBS supplemented with 0.5% bovine serum albumin (BSA).

Packed RBC (PRBC): Blood was drawn from six healthy donors according to the blood bank routine and stored in standard sterile bags (not leukoreduced) containing citrate phosphate dextrose (CPD). PRBCs were stored (in SAGM) at 4 °C in the Hadassah Hospital Blood Bank until their expiration date (42 days). As above, RBC was isolated, washed, and re-suspended in PBS supplemented by 0.5% of BSA.

#### 4.2.3. Preparation of RBC Membranes

Cells were lysed in sodium phosphate lysis buffer (5 mM sodium phosphate pH 8.0, 1 mM EGTA, 1 mM EDTA, and 1 mM PMSF) on ice for 10 minutes. Lysates were centrifuged (14,000 rpm for 10 min), and the supernatant was aspirated. We repeated the procedure three times to clean the sample from hemoglobin. After the third wash, a protease inhibitor cocktail (Sigma, St. Louis, MO, USA) was added. Samples were sent in dry ice for mass-spectrometry analysis.

To test the changes in the protein composition of RBC, we utilized mass-spectrometry analysis to cover the whole spectrum of proteins to monitor changes in integral proteins of the erythrocyte membrane. The presented research examined protein composition for RBCs with different aging statuses.

#### 4.2.4. Determination of RBC Membrane Protein Composition

The composition of the RBC membrane proteins and their relative level in the cell membrane was comprehensively determined using mass-spectroscopy [69,70]. Proteins were trypsin-digested following the in-solution digestion protocol. Peptides were then purified on C_18_ StageTips prior to their LC-MS analysis. Peptides were separated on an Easy-spray pepmap column using a water-acetonitrile gradient and the EasynLC1000 nanoHPLC. Peptides were electrosprayed into a Q-Exactive mass spectrometer (Thermo Scientific, Waltham, MA, USA) via the Easy-spray source. Peptides were analyzed using data-dependent acquisition, with the fragmentation of the top 10 proteins from each scan. Raw MS files were analyzed by MaxQuant using the Human Uniprot database. The false discovery rate was set to 1% at the protein and peptide levels. Mass spectrometry identified and quantified 752 proteins and their levels.

#### 4.2.5. Determination of RBC Deformability

**Figure 5:**
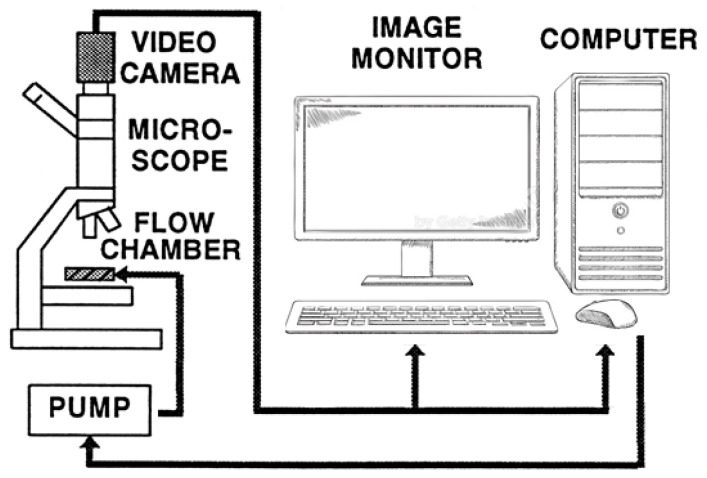
Scheme of the Cell Flow-properties Analyzer (CFA).

RBC deformability was determined using our original computerized cell flow-properties analyzer (CFA), as described in numerous previous studies [25,35,71,72], illustrated in Figure 5. The CFA provides the RBC deformability by direct visualization of the cell shape-change in a narrow-gap flow-chamber under flow-induced shear stress resembling conditions in microvessels. 

In brief, 50 μL of the RBC suspension (1% hematocrit, in PBS, supplemented by 0.5% BSA) was inserted into the flow chamber (adjusted to 200 μm gap) containing a glass slide, to which the RBC adhere, and the adherent RBC were then subjected to controllable flow-induced shear stress (3.0 Pa). The deformability is expressed by a change in cell shape expressed by the elongation ratio, ER = *a*/*b*, where “*a*” is the major cellular axis and “*b*” is the minor cellular axis. ER = 1.0 reflects a round RBC, undeformed by the applied shear stress. The CFA contains an image analysis program that automatically measures each cell’s ER. RBCs with ER ≤ 1.1 are defined as “undeformable” cells that do not deform under high shear stress. The image analysis produces an ER distribution in a population of 8000–10,000 cells, from which various parameters are derived, including the average ER (AER), the median ER (MER), the percent of undeformable cells (%UDFC, ER ≤ 1.1), and low-deformable cells (%LDFC, ER ≤ 1.3) [25,72].

#### 4.2.6. Statistical Analysis

The Shapiro–Wilk test was used to verify the normality of the distribution of the continuous variables. The results are presented as mean ± SD and tested for statistical significance using the paired Student t-test. Statistical differences, examined with the SPSS 21 (version 64 bit) software package, were considered significant at *p* < 0.05. The Pearson coefficient and p-value characterized the significance of linear regression between two tested parameters. The coefficient of variation (CV) was used to characterize the variability in the tested RBC samples’ cell deformability and protein level.

## 5. Conclusions

Red blood cell deformability, measured by image analysis, strongly correlates with the level of fourteen cell membrane proteins (“deformability-expressing proteins”), measured by mass spectroscopy.

The deformability-expressing proteins exhibit highly significant inter-correlations between their levels in the RBC membrane. These primarily include (1) proteins involved in the lipid bilayer-cytoskeleton linkage and (2) proteins involved in membrane lipid rafts. Notably, part of them are constituents of extracellular RBC micro-vesicles. This suggests that the decrease in deformability, as occurs during RBC physiological aging, is a programmed process of membrane remodeling and shedding membrane fragments, mirroring the formation of extracellular vesicles.

The highly significant inter-correlation between the deformability-expressing proteins infers that the cell deformability can be assessed by determining a few, or possibly one, of these proteins.

## Figures and Tables

**Table 1:** Correlations between the levels of RBC membrane proteins (expressed by Ln (LFQ)) and the cell deformability, expressed by the average elongation ratio (AER).

№	Proteins	Gene	Significance, *p*	Pearson Coefficient, r
1	Ezrin	EZR	0.00006	0.83
2	Long-chain-fatty-acid—CoA ligase 4	ACSL4	0.0002	0.81
3	Argonaute-2	EIF2C2	0.0003	0.79
4	Protein band 4.1	EPB41	0.0003	0.79
5	Glycophorin C	GYPC	0.0005	0.77
6	GTP-binding proteins Ras	RAC1/3	0.0007	0.76
7	Stomatin	STOM	0.0008	0.75
8	G-adducin	ADD3	0.001	0.75
9	Flotellin-1	FLOT1	0.001	0.73
10	CD44	CD44	0.002	0.72
11	Band-3	SLC4A1	0.002	0.72
12	Flotellin-2	FLOT2	0.002	0.71
13	Integrin-associated protein CD47	CD47	0.003	0.72
14	Glycophorins A	GYPA	0.004	0.67

**Table 2:** Correlation matrix for levels of deformability-expressing membrane proteins. Statistical significance expressed using Pearson correlation coefficient.

	EIF2C2	EZR	EPB41	ACSL4	FLOT1	GYPC	STOM	CD44	SLC4A1	FLOT2	RAC1/3	CD47	GYPA	ADD3
EIF2C2	1.00													
EZR	0.72	1.00												
EPB41	0.92	0.71	1.00											
ACSL4	0.90	0.81	0.86	1.00										
FLOT1	0.88	0.70	0.93	0.87	1.00									
GYPC	0.84	0.71	0.88	0.84	0.96	1.00								
STOM	0.85	0.71	0.85	0.91	0.94	0.96	1.00							
CD44	0.89	0.67	0.84	0.89	0.93	0.95	0.98	1.00						
SLC4A1	0.85	0.62	0.89	0.84	0.97	0.98	0.97	0.95	1.00					
FLOT2	0.85	0.67	0.84	0.89	0.96	0.97	0.99	0.98	0.98	1.00				
RAC1/3	0.85	0.72	0.84	0.91	0.93	0.94	0.97	0.95	0.95	0.97	1.00			
CD47	0.82	0.69	0.78	0.86	0.88	0.89	0.95	0.93	0.92	0.95	0.96	1.00		
GYPA	0.86	0.58	0.91	0.79	0.89	0.85	0.83	0.83	0.90	0.86	0.90	0.84	1.00	
ADD3	0.75	0.79	0.78	0.69	0.68	0.67	0.60	0.63	0.63	0.58	0.67	0.57	0.67	1.00

## Data Availability

Data can be available upon request.
